# The enhancement of medical student performance through narrative reflective practice: a pilot project

**Published:** 2013-03-31

**Authors:** Alan Thomson, Dwight Harley, Marie Cave, Jean Clandinin

**Affiliations:** 1 Department of Medicine, Western University, London, Ontario, Canada; 2 Division of Studies in Medical Education, University of Alberta, Edmonton, Alberta, Canada; 3 Department of Family Medicine, University of Alberta, Edmonton, Alberta, Canada; 4 Center for Research for Teacher Education and Development, University of Alberta, Edmonton, Alberta, Canada

## Abstract

**Background:**

Narrative Reflective Practice (NRP) is a process that helps medical students become better listeners and physicians. We hypothesized that NRP would enhance students’ performance on multiple-choice question exams (MCQs), on objective structured clinical examinations (OSCEs), and on subjective clinical evaluations (SCEs).

**Methods:**

The MCQs, OSCEs and SCEs test scores from 139 third year University of Alberta medical students from the same class doing their Internal Medicine rotation were collected over a 12 month period. All preceptors followed the same one-hour clinical teaching format, except for the single preceptor who incorporated 2 weeks of NRP in the usual clinical teaching of 16 students. The testing was done at the end of each 8-week rotation, and all students within each cohort received the same MCQs, OSCE and SCEs

**Results:**

Independent t-tests were used to assess group differences in the mean MCQ, OSCE and SCE scores. The group receiving NRP training scored 4.7% higher on the MCQ component than those who did not. The mean differences for OSCE and SCE scores were non-significant.

**Conclusions:**

Two weeks NRP exposure produced an absolute increase in students’ MCQ score. Longer periods of NRP exposure may also increase the OSCE and SCE scores. This promising pilot project needs to be confirmed using several trained preceptors and trainees at different levels of their clinical experience.

## Introduction

Professionals with years of experience know more than do novices to the profession, but what they know is not just in textbooks or journals. Experience generates knowledge that is narrative, tacit, expressed in practice, and formed in storied contexts. The nature of this knowledge is that it is difficult to teach in traditional pedagogical settings. However, reflection on practice, through the telling of stories of experience, enables professionals to construct storied accounts of their tacit knowledge. In this way, professional practice emerges as a reflective practice. The idea of reflective knowing-in-action that emerges from reflection on practice owes a great deal to the ideas of Dewey and his concept of experience and knowledge grounded in experience.^1^ Schon, following Dewey, highlighted the place of professional growth through various approaches to reflective practice.^2^–^4^ Further work by Clandinin and Connelly (1995) with the professional knowledge of teachers is the theoretical grounding for the research reported here on the professional growth of medical students.^2^,^5^

These theoretical underpinnings have resulted in a pedagogical approach called Narrative Reflective Practice (NRP). The central purpose of NRP in medicine is to improve the clinical skills of physicians and physicians in training, particularly their skills in communicating with, and listening to patients. NRP in medicine is focused on: physicians listening to and reflecting upon, patients’ stories; listening to physicians’ stories which include their medical agendas; hearing physicians’ stories of experience in order to hone those experiences, and, thereby, develop their personal professional knowledge and clinical expertise.

### Other approaches to reflective practice

Other medical educators approached reflection indirectly. Henderson and Johnson describe a course for medical students designed to encourage the development of professional identity, both within a workshop experience and later in a course evaluation and email communication with group facilitators.^6^ Henderson and Johnson, drawing on Schon’s work, designed a course for medical students to enable medical students to reflect in action, while the writing and dialogue with a facilitator promoted reflected-on action.^6^ Neither the workshop or course evaluations were tied to summative student evaluations. The success of this intervention is in contrast to another, where students were apprehensive of the required submissions of their written reflections around a critical incident or significant event analysis (SEA). Many of these interventions in medical education occurred during clerkship years.

Other interventions draw on more narrative notions of reflective practice. Instead of expecting learners to volunteer their experiences at the outset, they suggest that reflecting on the expression of others’ experiences in the humanities can trigger reflection. Charon encourages the use of literature as a way to encourage reflection.^7^ She believes that reading literature can help physicians understand the illness experience and to enhance clinical skills necessary in diagnosis, ethical clinical decision-making, and management of patients. She also stresses the value to the person of the physician that results from increasing self-awareness and providing meaning. Lazarus and Rosslyn^8^ also used literature as part of a special study module with medical students. The module’s aim was to use the study of the arts to enhance students’ understanding of the illness experience. One objective was to encourage medical students to reflect on, “how the experience has affected their own personal and professional development”. Other medical practitioners including Coulehan^9^ in Canada, and Greenhalgh and Collard^10^ in Britain, have also developed conceptualizations of narrative medicine. For example, Coulehan draws attention to the hidden curriculum in medical education, which emerges from the storied context of medical education. Coulehan asks that we attend to the hospital narratives, that is, the storied contexts of medical education and ask ourselves about the stories that surround physicians and physicians in training.^9^ Bolton uses several humanities-based methods to promote reflection in physicians at various stages of their professional development.^11^

### The intervention in medical education

There is general acceptance that medical trainees learn from preparing the history and physical (H & P) report on recently admitted patients who will be under their care. However, it is not clear if the enrichment of the process through formative feedback provides superior outcomes in terms of professional knowledge. It was decided to use a narrative reflective practice pedagogical strategy to ascertain if there was improvement in clinical skills and knowledge. A description of the strategy is provided below.

### The Context of the Study

Student Interns (medical students, year III) working in teams in Internal Medicine at the University of Alberta (U of A) rotate for eight weeks through one of four hospitals: the University of Alberta Hospital (UAH), the Royal Alexandra Hospital (RAH), the Grey Nuns Hospital (GNH), and the Misericordia Hospital (MIS). The training at these four Edmonton hospitals is on clinical teaching units (CTUs), in which there is a staff physician, one senior and several junior residents, as well as several third-year medical students.

There is no information available as to whether the objective outcome of the training such as multiple-choice questions is influenced by NRP. There are a number of outcomes that could be assessed. However, in order for administrators, clinician teachers and student trainees to accept the potential benefit of narrative reflective medicine, the evaluations needed to be in a form that is already in place or considered to be suitable and measurable within the context of the historical approaches to medical examination. We proposed firstly to use the anonymous results of the trainees’ OSCE (Observed, Standardized Clinical Examination), and the multiple-choice questions (MCQs) taken at the end of their internal medicine rotation to compare the group which did, and the groups which did not, have NRP during their Internal Medicine rotation on the CTUs at UAH, RAH, GNH and MIS. Finally, the standard evaluation and feedback forms prepared regularly by the CTU preceptors on each medical student during their rotation were used to compare the preceptors’ clinical performance.

The purpose of this study was to use the standard assessment tools for clinical training of third year medical students to determine the effect of narrative medicine on the trainee’s performance. The Null Hypothesis of this study was: “there is no effect of Narrative and Reflective Practice on medical student test scores on the end of rotation multiple-choice questions (MCQs), Observed Standardized Clinical Examinations (OSCEs), and the student rotation evaluations”.

## Methods

### Study Design

In the third year of their medical education curriculum, all University of Alberta (U of A) medical students requested which clinical teaching unit (CTU) of the four teaching hospitals they wished to be assigned; whenever possible these requests were honoured. With their preceptor, students interact with patients, learn to take a medical history, examine the patient, record the interaction, and present their findings orally and in writing. Under the direct supervision of a resident and the staff preceptor, the student assists in the care of 2–5 patients on the CTU.

At the UAH, the one NRP preceptor, over a time span of two weeks, met with 6–10 medical students for one hour each morning for 8 mornings. For the remaining 6 weeks of their CTU exposure at the UAH, students met with other preceptors. There was one preceptor for 6–10 students. This represents the standard teaching model, without the intervention of NRP. At the end of the students’ clinical experience, the “Narrative Reflective Practice” preceptor asked the students to write a brief account of their reflection on a patient’s experience of disease and illness, and how this experience affected the student. The task was for the students to hear the patient’s story. This usually included more details about the way in which the illness affected the lives of their patients, the beliefs of the patients about the cause and nature of the disease, their lives outside the domain of their medical history, their families, or their social history. The student then wrote a narrative account of his/her experience of how they were affected by the patient. The students volunteered to read their narratives aloud to the group. They were encouraged to explore themes such as their own response to these stories, the ways to deal with these feelings, the effect of these factors on the patient’s choice of, or response to, treatment. The student group members discussed each shared experience, and reflected upon similar experiences to which they had been exposed, and their own thoughts and feelings.

The study ran over 54 consecutive weeks. Each rotation was eight weeks in duration. The lead author at one institution (UAH) provided the Narrative and Reflective Practice (NRP). Some students rotating at UAH did not have the author as a preceptor and did not have NRP.

### Outcome Measures

The student’s confidential “PIN” was used to ensure that the students who had been part of this procedure did not have their identity released to the academic authorities. To ensure this, the administrator of the Division of Studies in Medical Education was responsible for coding the students’ identity, and providing the test results. At the end of each CTU rotation, each of the students’ preceptors provided a Subjective Clinical Evaluation (SCE) using a common evaluation form. The SCEs have not been validated. Students also took the same multiple-choice question (MCQ) examination, as well as the same Observed Standardized Clinical Examination (OSCE).

### Statistical Analysis

Descriptive statistics were calculated for the entire cohort as well as for each group individually. Differences across the three groups of students were tested for significance using a one-way analysis of variance (ANOVA). Differences between the intervention and the remaining cohort were tested using an independent t-test. All computations were done using SPSS and EXCEL.

## Results

The student’s SCE mark was not included in the analysis because there were numerous different preceptors at the four teaching hospitals, each group of students had different combinations of preceptors, and this method of evaluation had not been validated.

The MCQs covered a wide range of areas related to the diagnosis and treatment of disorders in, for example, the heart, lung and kidneys. These MCQs were drawn from a department bank that reflected the type of questions used in a final national examination. The OSCEs were also drawn from departmental/national questions.

The process of the OSCEs is uniform throughout Canada: the trainee is tested on 8 to 12 “stations”, each of which represents the testing of a clinical skill. For example, “examine the patient for liver disease.” This request is posted on the door of clinic examining room. The student reads the request; when a bell rings, she/he knocks on the door, enters the room, and introduces themselves to the patient or patient-actor. The student washes their hands, briefly explains what they plan to do, and then proceeds to perform inspection, palpation, percussion and auscultation of the appropriate body component relevant to the question/request.

During this process, an examiner sits quietly in the room, carefully watching the process, notes the student’s performance, and marks off predefined components of the clinical examination on a checklist. In this way, all students have the same question, same “patient”, same examiner, and same marking outline.

The MCQ and the OSCE tests were validated, and were identical for each eight-week group of students. These questions were drawn from the department’s bank of validated questions. The group means and standard deviations for the MCQ and OSCE measures are shown in Table 1.

The analysis compared the mean MCQ and OSCE scores among three groups of students: the NRP Intervention group, U of A - non-intervention groups, and students from the remaining three sites as a whole, who also represented a non-intervention group. A one-way ANOVA (Table 2) revealed that neither the MCQ score nor the OSCE score differed between the three groups of students. When comparing the mean MCQ score of 76.75% in the Intervention group against the other two groups combined, a significant difference was detected (p < 0.02). Students in the intervention group scored on average 4.7% raw score points higher. The group difference in OSCE score was not significant.

A correlation coefficient of 0.26 existed between the students’ OSCE and MCQ scores. This correlation is considered to be small (in terms of strength) but it suggests that the measures were assessing different knowledge/skill sets.

## Discussion

The study was designed to use a new pedagogical strategy (NRP) to better help medical trainees learn from preparing the history and physical (H & P) report on recently admitted patients who will be under their supervised care. Our purpose was to learn if such an approach would provide superior outcomes in terms of professional knowledge or skills.

We suggest that NRP is a way to develop the clinical skills of caring, compassion, and listening. The SCE did not assess these considerations. We did not assess patient satisfaction in this study. We wished to determine if NRP improves student performance scores on the two objective evaluation tools currently in use in the Faculty of Medicine, MCQs and OSCEs. NRP was found to improve the MCQ by 4.9%. We propose that the magnitude of this objective improvement is sufficient evidence to justify a larger study involving the training of teaching staff at each of the four teaching hospitals, and the provision of resources to introduce NRP into all clinical training experiences.

The time commitment for NRP training is approximately four hours, and a group of 12 preceptors can be instructed by a single trainer. For the preceptors who take the faculty development workshop, there will likely be an improvement in their own clinical skills as well as skill in using a new pedagogical approach. For the students, they gain increased skills in talking and listening to patients, in understanding the relationship of disease to other aspects of patient’s lives, and a way of adapting to the stress and personal emotional distress associated with patient care and becoming a physician.

We acknowledge that there are limitations to this study. This was a pilot project. Encouraged by the meaningful increase in the scores of students exposed to NRP (4.9%), we would wish to undertake a larger study using several preceptors trained in NRP. A longer duration of NRP training might demonstrate improvement in the student score in another objective measure, the OSCE. A follow-up of the NRP-exposed students from their third to fourth year would be useful to establish the durability of the NRP effect, and the undertaking of a multicentre study would help to establish the generalizability of the NRP improvement. Finally, in a future study we would wish to undertake a patient-focused inquiry into their satisfaction with the care provided by students with NRP versus without NRP.

## Conclusion

We do not foresee any adverse effects of the introduction of NRP. The results from third year students on CTUs may also be generalizable to other forms and levels of medical student teaching. For example, for the last three years, the NRP preceptor involved in this pilot study used NRP with second year medical students’ clinical skills teaching. The students receiving NRP for 15 hours did very much better on their OSCE examination, and on their final history and physical assignment.

## Figures and Tables

**Table 1:** Group means and standard deviations

Measure	Group	N	Mean*	Standard Deviation
**MCQ**	Intervention	16	76.75	8.62
	U of A	9	70.44	7.14
	Other	114	72.15	7.62
	Total	139	72.57	7.82

**OSCE**	Intervention	16	72.04	6.00
	U of A	9	74.86	5.22
	Other	114	72.86	5.86
	Total	139	72.90	5.82

* These values represent raw percentage scores on the MCQ and OSCE examinations. The only significant difference was between the Intervention (NRP) group versus the other centre non-NRP groups.

A small correlation existed between the MCQ and the OSCE grades (0.26, p < 0.001).

**Table 2:** ANOVA for between group differences

Variable	Source	SS	df	MS	F	Sig.
**MCQ**	Between Groups	340.41	2	170.21	2.86	0.06
	Within Groups	8091.69	136	59.50		
	Total	8432.10	138			

**OSCE**	Between Groups	46.83	2	23.42	0.69	0.50
	Within Groups	4631.23	136	34.05		
	Total	4678.06	138			
